# Adequately dosed aerobic physical activity in people with axial spondyloarthritis: associations with physical therapy

**DOI:** 10.1007/s00296-020-04637-x

**Published:** 2020-06-28

**Authors:** Bas Hilberdink, Thea Vliet Vlieland, Florus van der Giesen, Floris van Gaalen, Robbert Goekoop, Andreas Peeters, Marta Fiocco, Salima van Weely

**Affiliations:** 1 Department of Orthopaedics, Rehabilitation and Physical Therapy, Leiden University Medical Center, j11, P.O. Box 9600, 2300 RC Leiden, the Netherlands; 2 Department of Rheumatology, Leiden University Medical Center, Leiden, the Netherlands; 3 Haga Hospital, The Hague, the Netherlands; 4 Reinier de Graaf Gasthuis, Delft, the Netherlands; 5 Department of Biomedical Data Science, Medical Statistics, Leiden University Medical Center, Leiden, the Netherlands; 6 Mathematical Institute, Leiden University, Leiden, the Netherlands

**Keywords:** Axial spondyloarthritis, Physical activity, Exercise, Physical therapy

## Abstract

**Introduction:**

This study aimed to compare the engagement in moderate- and vigorous-intensity PA in axSpA patients with and without current physical therapy (PT).

**Methods:**

In this cross-sectional study, a survey, including current PT treatment (yes/no) and PA, using the ‘Short QUestionnaire to ASsess Health-enhancing PA’ (SQUASH), was sent to 458 axSpA patients from three Dutch hospitals. From the SQUASH, the proportions meeting aerobic PA recommendations (≥ 150 min/week moderate-, ≥ 75 min/week vigorous-intensity PA or equivalent combination; yes/no) were calculated. To investigate the association between PT treatment and meeting the PA recommendations, odds ratios (OR) with 95% confidence intervals (95% CI) were estimated using logistic regression models, adjusting for sex, age, health status and hospital.

**Results:**

The questionnaire was completed by 200 patients, of whom 68%, 50% and 82% met the moderate-, vigorous- or combined-intensity PA recommendations, respectively. Ninety-nine patients (50%) had PT treatment, and those patients were more likely to meet the moderate- (OR 2.09 [95% CI 1.09–3.99]) or combined-intensity (OR 3.35 [95% CI 1.38–8.13]) PA recommendations, but not the vigorous-intensity PA recommendation (OR 1.53 [95% CI 0.80–2.93]). Aerobic exercise was executed in 19% of individual PT programs.

**Conclusion:**

AxSpA patients with PT were more likely to meet the moderate- and combined-intensity PA recommendations, whereas there was no difference in meeting the vigorous-intensity PA recommendation. Irrespective of having PT treatment, recommendations for vigorous-intensity PA are met by only half of the patients. Implementation should thus focus on aerobic PA in patients without PT and on vigorous-intensity PA in PT programs.

## Introduction

Axial spondyloarthritis (axSpA) is a chronic inflammatory rheumatic disease, with back pain and stiffness as main symptoms and encompassing both non-radiographic and radiographic axSpA (ankylosing spondylitis) [1, 2]. The literature shows that axSpA is associated with both decreased cardiorespiratory fitness [3–6] and an increased risk of cardiovascular disease [7, 8], which are interrelated [9–12]. Adequately dosed aerobic physical activity (PA) according to public health recommendations improves cardiorespiratory fitness in people with axSpA [13, 14] and might reduce the cardiovascular risk. For this reason, it is advocated in international recommendations on PA in people with rheumatic and musculoskeletal conditions [15]. Aerobic PA concerns PA executed with moderate or vigorous intensity. Recent studies suggest that vigorous-intensity PA is most effective in improving cardiorespiratory fitness and reducing cardiovascular risk [10, 16–18] and it shows to be both beneficial and safe for people with axSpA [19, 20]. Therefore, especially vigorous-intensity PA should be pursued by people with axSpA, at least by those without an increased risk of cardiovascular complications during exercise.

This raises the question to what extent people with axSpA are actually engaged in aerobic PA, either or not with vigorous intensity. A previous study reported that evidence on PA engagement of people with axSpA is limited and heterogeneous in nature [3]. Nevertheless, it appears that the engagement in adequately dosed aerobic PA is insufficient, in particular in vigorous-intensity aerobic PA [21–24]. Three studies, all using accelerometers, showed that people with axSpA engaged less in vigorous-intensity PA than population controls, while the total amount of PA was comparable [21–23]. Another study found that more people with axSpA comply with the moderate-intensity PA recommendation (57%) than with the vigorous-intensity PA recommendation (32%), using a non-validated PA questionnaire [24]. That study used the PA recommendation prescribing moderate-intensity PA for ≥ 30 min on ≥ 5 days per week or vigorous-intensity PA for ≥ 20 min on ≥ 3 days. Other studies on aerobic PA in people with axSpA [3, 21, 23, 25] were based on the recommendation by the World Health Organization (WHO) [26], which does not state a required minimum frequency, but prescribes ≥ 150 min of moderate-intensity PA, ≥ 75 min of vigorous-intensity PA per week or an equivalent combination of this. It was reported that this recommendation was met by approximately half of patients [21, 23, 25], but no distinction was made between the proportions of people meeting the moderate- or vigorous-intensity PA recommendations. None of the studies distinguished between leisure time and work-related aerobic PA either, whereas leisure time PA appears to have greater health benefits [27–31] and is probably more easily modifiable than work-related PA. This superiority of leisure time PA could probably be caused by the difference in the nature of activities or by more opportunities to rest when desired and recover between sessions [27, 29].

Another limitation of previous studies on aerobic PA among people with axSpA, besides not distinguishing between moderate- and vigorous-intensity PA and between leisure time and work-related aerobic PA, is that none of the studies so far took the role of physical therapy (PT) into account. This is striking as relatively many axSpA patients have PT treatment [32] and it is generally acknowledged that apart from other health professionals, physical therapists play an important role in the promotion of PA [15]. However, it appears that aerobic PA may not be included in PT treatments often [32] and that the aerobic PA employed in exercise programs for people with axSpA is often inadequately dosed [20, 33–35].

To implement aerobic PA recommendations in people with axSpA, it is important to know what the focus of implementation activities should be, both in patients with and without PT treatment. Due to the physical limitations for which axSpA patients seek PT treatment, it is not necessarily expected that patients with PT are more inclined to meet the aerobic PA recommendations. Moreover, PT programs may not include (advice on) aerobic PA [32]. Given the lack of knowledge on the association between having PT treatment and meeting aerobic PA recommendations among people with axSpA, the aim of the present study was to compare the engagement in moderate- and vigorous-intensity PA (during work and leisure time) in axSpA patients with and without PT treatment.

## Methods

### Study design and setting

This cross-sectional, multicenter study consisted of a once-only survey among people with axSpA living in the southwestern region of the Netherlands. In this survey, participants were asked whether they had either individual or group PT treatment, to compare PA of patients with and without any guidance from a physical therapist. In the Netherlands, PT for people with axSpA can both be offered on an individual basis or by means of axSpA-specific supervised group exercise. This group exercise usually consists of weekly land- and water based exercises supervised by a physical therapist and is organized by local patient associations for people with a rheumatic disease [34]. The study obtained ethical approval from the Leiden University Hospital Ethical committee (P14.326). The reporting of this study was done in accordance with the checklist for cross-sectional studies from the ‘Strengthening the Reporting of Observational Studies in Epidemiology (STROBE) Statement’.

### Patients

In 2015, registers of three hospitals in the southwestern region of the Netherlands (Leiden University Medical Center in Leiden, Haga Hospital in The Hague and Reinier de Graaf Gasthuis in Delft) were screened for patients with a confirmed diagnosis of axSpA who had ever visited the rheumatology outpatient clinic. The survey was sent by postal mail to eligible patients, including an invitation letter on behalf of their treating rheumatologist, an information leaflet, an informed consent form and a pre-stamped envelope. No reminders were sent.

### Assessments

The survey was self-developed and first pilot-tested by patient representatives affiliated with the Dutch Arthritis Society. It measured the following variables:

- Demographic and clinical characteristics: sex, age, year of diagnosis and use of medication related to axSpA (painkillers (acetaminophen or opioid painkillers); Non-Steroidal Anti-Inflammatory Drugs (NSAIDs), biological Disease-Modifying Antirheumatic Drugs (DMARDs); synthetic DMARDs; no medication related to axSpA).
- Health status, using the Assessment of Spondyloarthritis International Society Health Index (ASASHI), which is a valid, reliable and responsive questionnaire measuring functioning, health and disease impact in people with axSpA [36, 37]. The ASAS HI includes 17 questions and results in a score between 0 and 17, with a lower score indicating a better health status.
- PT treatment, by asking whether they had PT treatment, either at the time the study was conducted (*current PT*; yes/no) or ever in the past (yes/no). Moreover, it was asked whether they were or had been treated individually in a practice (yes/no) and/or in a group with axSpA-specific group exercise therapy (yes/no). Furthermore, for individual PT, the duration (> 5 years, > 3 years, > 1 year, > 6 months or < 6 months), frequency (less than weekly, weekly, twice weekly, more than twice weekly) and contents (15 treatment options) of PT treatment were recorded. These 15 treatment options were clustered according to the four groups of treatment modalities as described in the national physical therapists’ professional profile developed by the Royal Dutch Society of Physical Therapy [38]: Counseling (including education on home exercise; coping; and PA and sports); Exercise (including active joint range of motion exercises; muscle strengthening exercises; aerobic exercises; balance exercises; and relaxation exercises); Manual treatment (including passive mobilization; and massage); and Applying physical modalities (including thermotherapy; kinesiotaping; electrotherapy; ultrasound; and dry needling).
- Aerobic physical activity, using the validated Dutch version of the ‘Short QUestionnaire to ASsess Health-enhancing PA’ (SQUASH) [39, 40]. The SQUASH consists of 17 items asking respondents to recall PA as performed during a regular week in the past 12 months, yielding the time duration per PA intensity and the type of aerobic PA. The SQUASH categorizes PA into PA during commuting, (light and heavy) work, (light and heavy) household, walking, cycling, gardening, odd jobs and sports. For the purpose of this study, these categories were dichotomized into leisure time PA, including recreational walking, recreational cycling, exercise and sports, and non-leisure time PA, which includes PA during commuting, work, household, gardening and odd jobs. Using the compendium of Ainsworth [41], a research assistant (JP) assigned the correct MET-values to the corresponding activities. The SQUASH uses a syntax to categorize the activities into light-, moderate- and vigorous-intensity PA, by combining activities’ MET-values with both participants’ age and a subjective effort-score (slow, average, fast) that participants assigned to each activity. Aerobic PA includes all PA performed with at least moderate-intensity. The SQUASH data were used to calculate whether patients met the moderate- (≥ 150 min/week), vigorous- (≥ 75 min/week) and/or combined-intensity (≥ 75 min/week vigorous- and/or ≥ 150 min/week moderate- or vigorous-intensity PA) aerobic PA recommendations by the WHO [26]. This was examined both for PA during all daily activities and during leisure time specifically.

### Statistical analyzes

The returned questionnaires were scanned and analyzed by Cardiff® Software (California, United States) and manually checked and corrected afterwards. Descriptive statistics were used to describe patient characteristics, the proportions meeting the aerobic PA recommendations and the engaged types of leisure time and non-leisure time aerobic PA. This was done for the total group of participants and for patients with and without PT guidance separately. Results were reported as percentages or medians with minimum (Min) and maximum (Max) values, where appropriate.

To investigate the differences in characteristics between patients with and without PT, the median test for independent samples was used for continuous data and Pearson’s chi-square test for categorical data. In addition, six logistic regression models were estimated with meeting the moderate, vigorous or combined-intensity PA recommendations, both during all daily activities and during leisure time, as the dependent variables and current PT treatment (individual and/or group) as independent variable. To control for confounding, sex, age, health status and hospitals were included in the models as independent variables. All statistical analyzes were performed with IBM SPSS Statistics for Windows, version 23.0 (IBM Corp., Armonk, N.Y., USA).

## Results

### Patients

The questionnaire was sent to 458 axSpA patients of whom 206 returned it (response rate 45%). Six of them were excluded because the SQUASH data were either missing (*n* = 3) or invalid (*n* = 3).

Patient characteristics are presented in Table 1, for the total group and for patients with and without PT separately. The majority of patients was male (69%), the median age 57 years and the median disease duration 23 years. The median ASAS HI score was 5.3, indicating moderate health status [37]. Ninety-nine patients had PT treatment at the time the study was conducted: 77 had individual PT treatment in a private practice only, 11 participated in axSpA-specific group exercise therapy only and 11 had both individual PT treatment in a private practice and group exercise therapy (on two different days). The group exercise therapy consisted of a standardized program comprising weekly land- and water based mobility and strengthening exercises and sports (mostly volleyball) in most patients [34]. Table 2 shows the duration, frequency and contents of current individual PT treatment. Among the 88 participants who were receiving individual PT at the time the study was conducted, the duration of treatment was more than five years in 66 patients (75%) and the treatment took place less than once a week in 44 patients (50%). Furthermore, the individual PT treatment included counseling in 67 (76%), exercise in 47 (53%), manual treatment in 80 (91%) and the application of physical modalities in 24 (27%). Regarding contents with a direct link to aerobic PA recommendations, education on PA and sports was reported by 37 patients (42%) and aerobic exercise during PT treatment by 17 (19%). Among the 101 participants without current PT, 84 had PT treatment ever in the past. No statistically significant differences regarding sex, age, disease duration, medication use, ASAS HI score and being employed were found between patients with and without PT.

**Table 1:** Differences in characteristics between axial spondyloarthritis patients with (*n* = 99) and without (*n* = 101) current physical therapy (PT), participating in a survey on physical activity and PT

	Total group (*n* = 200)	With PT (*n *= 99)	Without PT (*n* = 101)	*p*-value*
Sex, male, *n* (%)	138 (69)	70 (71)	68 (68)	0.679
Age, years, median (Min–Max)	57 (23–93)	59 (23–85)	54 (23–93)	0.066
Disease duration, years, median (Min–Max)	23 (1–58)	25 (1–58)	17 (2–58)	0.127
Medication use, *n* (%)				
Painkiller^a^	78 (39)	42 (42)	36 (36)	0.326
NSAID	123 (62)	64 (65)	59 (58)	0.365
Biological DMARD	77 (39)	39 (39)	38 (38)	0.797
Synthetic DMARD	25 (13)	11 (11)	14 (14)	0.557
None	16 (8)	5 (5)	11 (11)	0.128
ASAS HI score, median (Min–Max)	5.3 (0–14.9)	6.0 (0–13.4)	5.0 (0–14.9)	0.669
Being employed,* n* %	110 (55)	55 (56)	55 (54)	0.990

*P-value of chi-square test (for nominal variables) or median test (for continuous variables) for differences between patients with and without PT

^a^Acetaminophen or opioid painkillers

*Min *minimum value, *Max *maximum value, *NSAID *non-steroidal anti-inflammatory drugs, *DMARD *disease-modifying antirheumatic drugs, *ASAS HI *assessment of spondyloarthritis international society health index

**Table 2:** Duration, frequency and contents of individual physical therapy (PT) in people with axial spondyloarthritis (axSpA) participating in a survey on physical activity and PT (*n* = 88)

	AxSpA patients with individual PT (*n* = 88)
PT duration, *n* (%)	
> 5 years	66 (75)
3–5 years	8 (9)
1–3 years	5 (6)
6 months-1 year	4 (5)
< 6 months	5 (6)
PT frequency, *n* (%)	
More than twice weekly	0
Twice weekly	13 (15)
Weekly	30 (34)
Less than weekly	44 (50)
PT contents, *n* (%)	
Counseling	67 (76)
Education on home exercise	54 (61)
Education on coping	31 (35)
Education on physical activity and sports	37 (42)
Exercise	47 (53)
Active joint range of motion exercises	28 (32)
Muscle strengthening exercises	36 (41)
Aerobic exercises	17 (19)
Balance exercises	11 (13)
Relaxation exercises	3 (3)
Manual treatments	80 (91)
Passive mobilization	62 (71)
Massage	50 (57)
Physical modalities	24 (27)
Thermotherapy	9 (10)
Kinesiotaping	2 (2)
Electrotherapy or ultrasound	16 (18)
Dry needling	4 (5)

### Aerobic PA recommendations

Table 3 presents the proportions of participants meeting the aerobic PA recommendations during all daily activities and during leisure time. This table shows that for all daily PA, the moderate, vigorous- and combined-intensity PA recommendations were met by 68%, 50% and 82% of the participants, respectively. With respect to meeting the aerobic PA recommendations by taking only leisure time PA into account, the proportions of participants meeting the moderate-, vigorous- and combined-intensity PA recommendations were 48%, 42% and 67%, respectively. Moreover, 68% of the participants engaged in any moderate-intensity leisure time activities, whereas 50% of participants engaged in any vigorous-intensity leisure time activities.

**Table 3:** Differences in meeting combined-, moderate- and vigorous-intensity physical activity (PA) recommendations during all daily activities and during leisure time between axial spondyloarthritis patients with (*n* = 99) and without (*n* = 101) current physical therapy (PT), participating in a survey on physical activity and PT

	Total group (*n* = 200)	With PT (*n* = 99)	Without PT (n = 101)	OR*	95% CI
Meeting combined-intensity PA recommendation					
With all daily PA, *n* (%)	164 (82)	88 (89)	76 (75)	**3.35**	**1.38**–**8.13**
With leisure time PA, *n* (%)	133 (67)	72 (73)	61 (60)	1.81	0.94–3.49
Meeting moderate-intensity PA recommendation					
With all daily PA, *n* (%)	136 (68)	74 (75)	62 (61)	**2.09**	**1.09**–**3.99**
With leisure time PA, *n* (%)	96 (48)	55 (56)	41 (41)	**1.86**	**1.03**–**3.36**
Meeting vigorous-intensity PA recommendation					
With all daily PA, *n* (%)	100 (50)	54 (55)	46 (46)	1.53	0.80–2.93
With leisure time PA, *n* (%)	84 (42)	42 (42)	42 (42)	1.01	0.53–1.90

*Odds ratio adjusted for sex, age, health status and affiliated hospitals using multivariate logistic regression models

*OR *odds ratio. *CI *Confidence Interval. All daily PA = PA during commuting, household, work, gardening and odd jobs and leisure PA. Leisure time PA = recreational walking and cycling, exercise and sports. Combined-intensity PA recommendation = 150 min/week at least moderate-intensity PA or 75 min/week vigorous-intensity PA. Moderate-intensity PA recommendation = 150 min/week moderate-intensity PA. Vigorous-intensity PA recommendation = 75 min/week vigorous-intensity PA

### PT and aerobic PA recommendations

**Fig. 1:**
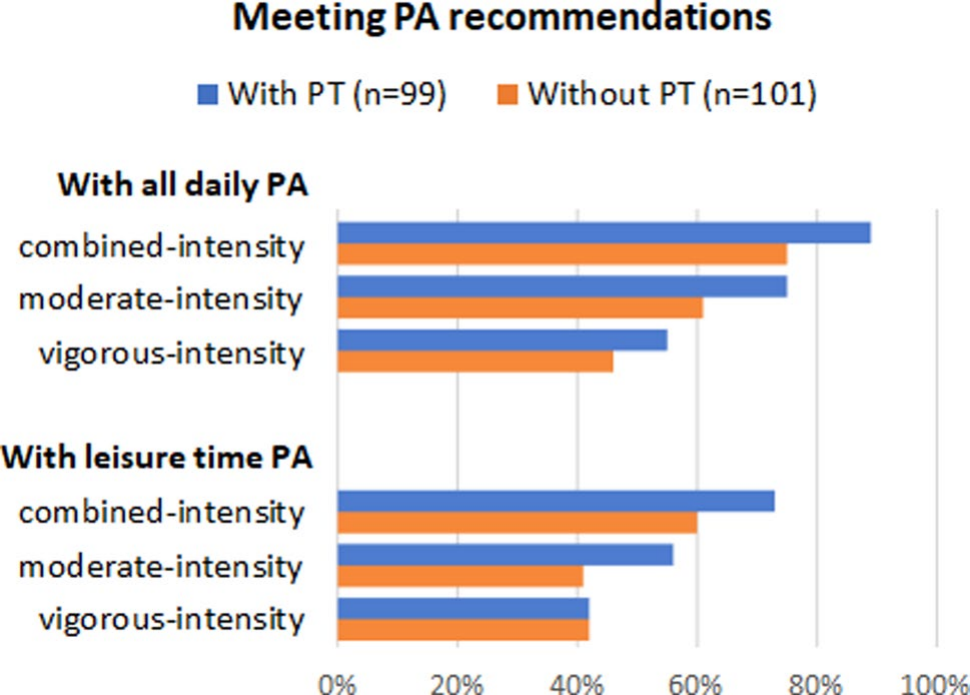
Proportions of axSpA patients with and without physical therapy (PT) meeting the combined-, moderate- and vigorous-intensity PA recommendations, both when including all daily PA and when only including leisure time PA

To study the association between PT treatment and aerobic PA, only current PT treatment was considered, since almost all participants (92%) had ever had PT. The differences between patients with and without current PT regarding the meeting of aerobic PA recommendations are shown in Fig. 1 and Table 3. Table 3 shows that, considering all daily PA, patients with PT are significantly more likely to meet the moderate- (OR 2.09 [95% CI 1.09–3.99]) and combined-intensity (OR 3.35 [95% CI 1.38–8.13]) PA recommendations than patients without current PT after adjusting for sex, age, health status and hospital. When only including leisure time PA, patients with PT are more likely to meet the moderate-intensity PA recommendation (OR 1.86 [95% CI 1.03–3.36]) than patients without PT, with no differences for the vigorous- or combined-intensity PA recommendations.

### Types of aerobic activities

**Fig. 2:**
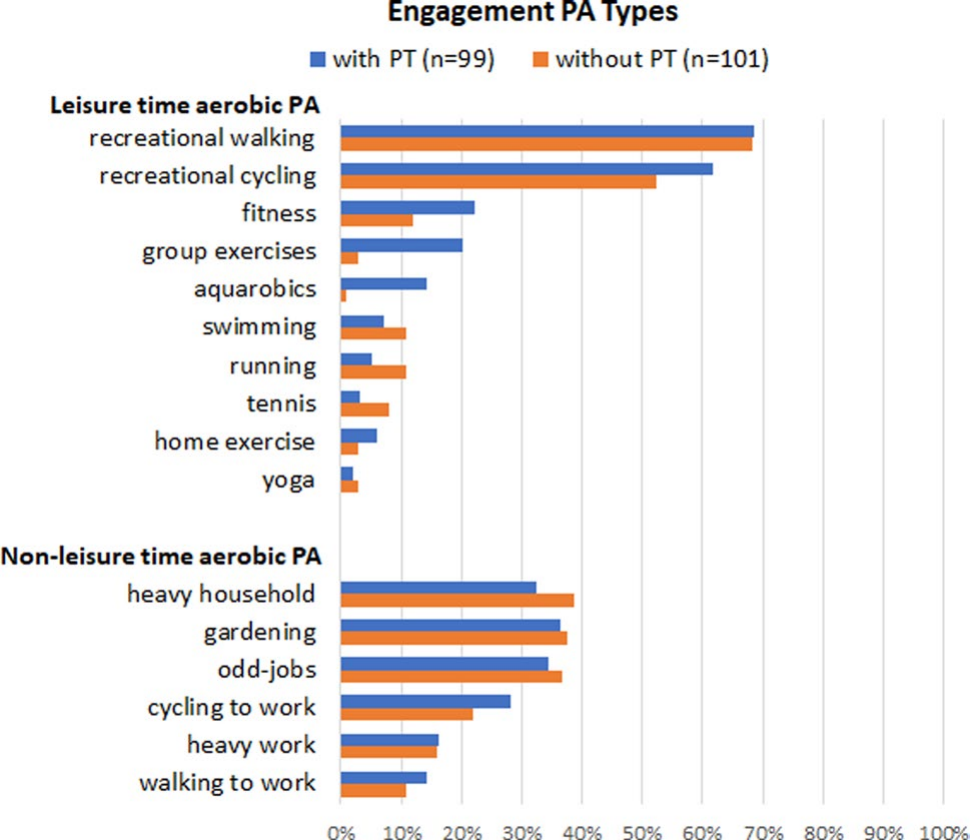
Proportions of axSpA patients with and without physical therapy (PT) engaging in different forms of leisure time aerobic PA (with > 2% patients participating) and other aerobic PA types

Figure 2 presents the proportions of axSpA patients with and without PT engaging weekly in different forms of leisure or non-leisure time aerobic PA. There were no statistically significant differences between the proportions of participants with and without PT engaging in the different types of aerobic activities, besides engagement in group exercise and aqua-aerobics; these types of aerobic PA were executed by significantly more patients with PT. This difference is likely to be due to participants with group PT, which often consists of group exercise and hydrotherapy in the Netherlands [34]. In both groups, it appeared that walking (69%) and cycling (57%) were the most frequently performed aerobic activities.

## Discussion

This study found that people with axSpA who were having PT treatment were more likely to meet the moderate- and combined-intensity aerobic PA recommendations than those without PT, whereas there were no differences in meeting the vigorous-intensity PA recommendation. Irrespective of current PT treatment, the proportion of participants meeting the vigorous-intensity PA recommendation was relatively low and often not attained with leisure time activities.

The finding that having PT treatment was associated with meeting aerobic PA recommendations was not necessarily expected, because PT programs may not include aerobic PA and those who need PT treatment are expected to have more physical limitations and may thus be less physically active. In our study, PT treatment was related to more aerobic PA, but this did not pertain to vigorous-intensity PA. Given the cross-sectional design of the study, it remains unclear whether the association between PT and aerobic PA is a result of PT treatment or that axSpA patients who are already relatively active are more inclined to seek PT treatment. Either way, the findings show that specifically axSpA patients without PT should be better educated on the benefits of aerobic PA. It is recently recommended that all health professionals in rheumatology should promote aerobic PA [15], but especially physical therapists could play an important role in such education, in particular since most individuals with axSpA have PT treatment at some point during their disease course, as confirmed in the present study. However, there is room for improvement in those with PT as well. Our study showed that education on PA is currently only provided in 42% of axSpA patients with individual PT in the Netherlands. In addition, aerobic exercise was only executed during PT in 19% of individual PT programs. This is unfavorable, as guided practice is one of the most important intervention components to optimize exercise behavior of axSpA patients [42]. Ideally, axSpA patients could experience and practice vigorous-intensity PA under supervision of a physical therapist. Therefore, aerobic PA should be included more often in individual PT programs, in particular with vigorous intensity.

The finding that particularly vigorous-intensity PA was performed insufficiently by relatively many axSpA patients is consistent with previous findings [21–24]. Similar to patients without PT, only half of those with PT met the vigorous-intensity PA recommendation. This finding could be related to results from previous studies, showing that appropriately dosed aerobic PA is often not included in (PT) exercise programs [33, 34]. A recent study on content of PT in axSpA patients found that in the Netherlands, aerobic exercises are only performed during individual PT in 22% of patients [32]. Hence, when implementing vigorous-intensity PA among people with axSpA, barriers and facilitators of both patients and therapists should be accounted for. A cross-sectional study examining these barriers and facilitators [19] found that motivation, disease symptoms and group heterogeneity could act as both barriers and facilitators according to patients and physical therapists. An implementation strategy could include education for therapists on how to motivate patients for vigorous-intensity PA and how to tailor and adjust it to varying symptoms, individual preferences and other potential variances among individual patients, such as the presence of comorbidity.

An important note when implementing vigorous-intensity PA is that caution is needed with sedentary individuals and people with an increased risk of cardiovascular complications during exercise [17, 43, 44]. Still, for most axSpA patients, vigorous-intensity PA should ultimately be aimed for, since this appears to have more health benefits [10, 16–18] and is more time-efficient [45], while time is an important exercise barrier in axSpA [19, 46, 47].

Regarding the types of actual activities, about half of the participants did not engage in any vigorous-intensity PA during leisure time at all. Studies reporting on the superiority of leisure time PA suggest that possible explanations for the greater benefits of leisure time PA are the difference in nature of activities and the presence of more opportunities to rest and recover when needed [27, 29]. The observation that recreational walking and cycling were the most popular forms of aerobic PA in our study could guide physical therapists in their advice and guidance on specific activities that are likely to be maintained in daily life. It is nevertheless conceivable that preferences for recreational activities may vary not only among individuals but among countries as well.

Overall, the proportion of patients meeting the WHO PA recommendation in the current study was much higher than in previous studies, namely 82% as opposed to around 50% in previous studies [21, 23, 25]. It is conceivable that the discrepancy might be due to the use of the SQUASH questionnaire. Another recent Dutch study using the SQUASH questionnaire among the general population and people with osteoarthritis found even slightly higher proportions of participants meeting the combined-intensity PA recommendation [48]. Nevertheless, despite the probable overestimation of the amount of aerobic PA, the current results are useful to compare subgroups within a population; the SQUASH has indeed shown to be fairly valid and reliable for within group comparisons [39, 40, 49]. Therefore, the SQUASH can be regarded as a valid measure to investigate the main objective of this study; to compare moderate- and vigorous-intensity PA between axSpA patients with and without PT. This comparison appears to not have been studied before and is important information to account for when implementing the aerobic PA recommendation.

This study has a number of limitations. First, because of the cross-sectional study design, no conclusions can be drawn about any causal relationships between having PT and aerobic PA. Second, and as already addressed, using a self-report questionnaire the amount of PA might have been overestimated [49]. Another limitation of the SQUASH is that it does not measure sedentary time. Moreover, it asks participants to recall their PA during a regular week in the past twelve months, whereas the groups compared in this study are based on having PT treatment at the time the study was conducted. As 89% of participants with individual PT were treated for more than 12 months, possibly not in all patients with PT, but at least in most of them, the actual influence of PT treatment on PA have been measured. Finally, the generalizability of our study is limited because the response rate was moderate (45%) and patients were recruited from only three hospitals in one region of the Netherlands. Although the participants of this study were relatively old [3], their sex ratio [3] and the proportion with PT [50] were comparable to other studies.

In conclusion, axSpA patients with PT were more likely to meet the moderate- and combined-intensity but not the vigorous-intensity aerobic PA recommendations than those without PT. These findings imply first of all that in axSpA patients without PT, aerobic PA must be promoted. Second, as vigorous-intensity PA appears insufficiently implemented among those with PT, additional education of physical therapists regarding the importance of and requirements for vigorous-intensity exercise as an essential element of PT programs for axSpA patients seems warranted. With the education of physical therapists, it should be noted that only 19% of patients with PT reported executing aerobic exercise as part of their PT treatment. This may indicate that there is a window of opportunity for physical therapists to increase patients’ engagement with vigorous-intensity PA. Future research should thus focus on interventions to optimize aerobic PA in axSpA patients without PT and on the implementation of vigorous-intensity exercise in PT programs.

## Related congress abstract publication:

We presented the data as a poster at the Annual European Congress of Rheumatology (EULAR 2019) and an abstract has therefore been published: Hilberdink, B., Van der Giesen, F., Vliet Vlieland, T., Van Gaalen, F., Ronday, K., Peeters, A., Van Weely, S. (2019). FRI0376 Differences in physical activity between axial spondyloarthritis patients with and without physical therapy. *Annals of the Rheumatic Diseases*, 78, 870–871.
